# The spike gating flow: A hierarchical structure-based spiking neural network for online gesture recognition

**DOI:** 10.3389/fnins.2022.923587

**Published:** 2022-11-02

**Authors:** Zihao Zhao, Yanhong Wang, Qiaosha Zou, Tie Xu, Fangbo Tao, Jiansong Zhang, Xiaoan Wang, C.-J. Richard Shi, Junwen Luo, Yuan Xie

**Affiliations:** ^1^School of Microelectronics, Fudan University, Shanghai, China; ^2^Alibaba DAMO Academy, Shanghai, China; ^3^Alibaba Group, Hangzhou, China; ^4^BrainUp Research Laboratory, Shanghai, China; ^5^Department of Electrical and Computer Engineering, University of Washington, Seattle, WA, United States

**Keywords:** spiking network, few-shot learning, online learning, gesture recognition, brain-inspired computation

## Abstract

Action recognition is an exciting research avenue for artificial intelligence since it may be a game changer in emerging industrial fields such as robotic visions and automobiles. However, current deep learning (DL) faces major challenges for such applications because of the huge computational cost and inefficient learning. Hence, we developed a novel brain-inspired spiking neural network (SNN) based system titled spiking gating flow (SGF) for online action learning. The developed system consists of multiple SGF units which are assembled in a hierarchical manner. A single SGF unit contains three layers: a feature extraction layer, an event-driven layer, and a histogram-based training layer. To demonstrate the capability of the developed system, we employed a standard dynamic vision sensor (DVS) gesture classification as a benchmark. The results indicated that we can achieve 87.5% of accuracy which is comparable with DL, but at a smaller training/inference data number ratio of 1.5:1. Only a single training epoch is required during the learning process. Meanwhile, to the best of our knowledge, this is the highest accuracy among the non-backpropagation based SNNs. Finally, we conclude the few-shot learning (FSL) paradigm of the developed network: 1) a hierarchical structure-based network design involves prior human knowledge; 2) SNNs for content-based global dynamic feature detection.

## 1. Introduction

Deep learning (DL) nowadays exerts a substantial impact on a wide range of computer vision tasks such as face recognition (Hu et al., 2015) and image classifications (Krizhevsky et al., 2012). But, it is still facing major challenges when processing information with high dimensional spatiotemporal dynamics such as video action recognition. This is because of two reasons: 1) the huge computational cost: the deep neural networks have to capture dynamic information across timing dimensions, which requires significant computational resources for the training stage (He et al., 2016) and 2) inefficient learning: events contain significant global dynamic features that are seldom captured by the DL, but these can be easily recognized by biological systems (Purves et al., 2014). One promising technology of sparsity (Liu et al., 2015, 2021; Wen et al., 2016) can relieve the first issue of the intensive computation to some extent, but the training cost is still enormous. Recently, few-shot learning (FSL) is proposed to tackle this problem. With prior knowledge, FSL can quickly generalize to new tasks with only a few labeled training samples (Sung et al., 2018; Wang et al., 2020; Chen et al., 2022).

Fortunately, spiking neural networks (SNNs) are an alternative candidate to perform spatiotemporal related tasks (Lobo et al., 2020) with FSL capability. By taking the natural characters of computing with time and in an event-driven manner, real-world event information will be encoded into spike trains as inputs along with timing frames. With a brain-inspired hierarchical network processing, the output spike patterns are interpreted as inference results *via* neural decoding methods (e.g., spike timing coding, rank coding, and spike count coding). Therefore, this could be an efficient technique for such applications, and another potential path toward the next generation AI (Furber and Temple, 2008).

However, employing SNNs for action recognition remains challenging since it lacks an efficient learning algorithm. Recently, SNN-based learning systems can be classified into three levels: a micro-level, a middle-level, and a macro-level system. A micro-level system emphasis place on utilizing low-level spiking neuron computing characters such as the temporal process and in an integration-and-fire manner (Caporale and Dan, 2008; Lee et al., 2016; Amir et al., 2017; Wu et al., 2018; Zhang and Li, 2019). For instance, Wu et al. (2018) proposed an SNN-based spatiotemporal back-propagation (BP) for a dynamic N-MINST event classification, the developed algorithm successfully combined spatial and temporal domain kernels and achieved inference accuracy of 98.78%. Also, Amir et al. (2017) illustrated a convolution neural network (CNN)-based SNN for gesture classification. By employing an event-driven sensor and the TrueNorth neuromorphic chip, the system shows 178.8 mW power consumption and 96.49% of accuracy. Meanwhile, a reservoir layer-based SNN utilizes the spike-timing dependent plasticity (STDP) rule to update weights. Such a novel network can achieve top-3 accuracy of 95% on IBM DVS gesture task (Amir et al., 2017). However, the higher-level computing entities in SNN such as attractor dynamics are missing in the system, which results in inefficient learning.

A middle-level system indicates that SNNs apply global dynamic behaviors in the learning process (Eliasmith, 2005; Sussillo and Abbott, 2009; Bekolay et al., 2014; Voelker et al., 2019; Luo and Chen, 2020; Chilkuri et al., 2021). A FORCE learning method (Sussillo and Abbott, 2009) is able to convert network chaos patterns into a desired one by modifying synaptic weights. Also, a neural engine framework (NEF) develops a method to build dynamic systems based on spiking neurons (Bekolay et al., 2014). Such an approach leverages neural non-linearity and weighted synaptic filters as computational resources. Compared to the first SNN type, a middle-level based SNNs place emphasis on global network dynamics rather than individual spiking neurons characters. Therefore, such systems demonstrate much better learning behaviors regarding scalability (Voelker et al., 2019) and model sizes (Chilkuri et al., 2021) in some particular scenarios.

A macro-level system includes advantages of both micro-level and middle-level systems (Sussillo and Abbott, 2009; Imam and Cleland, 2019). It combines detailed spiking neuron characters and network dynamics together to form a unique learning system. For instance, an olfactory SNN is largely based on the mammalian bulb network architecture, and with a line attractor-based neural plasticity rule for online learning odorants (Imam and Cleland, 2019). The developed system shows great one-shot learning behavior compared to the DL. Meanwhile, Wu et al. (2022) proposed a spike-based hybrid plasticity model for solving FSL, continual learning, and fault-tolerance learning problems, and it combines both local plasticity and global supervise information for multi-task learning.

**Figure 1:**
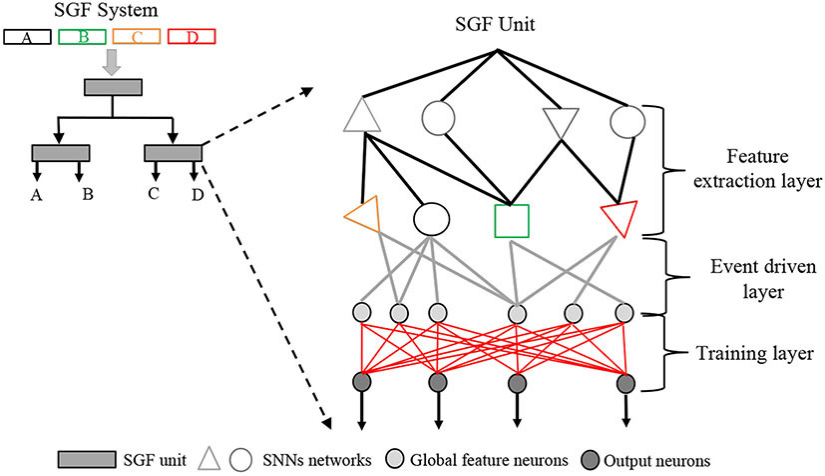
The spike gating flow (SGF) system concept. It consists of multiple SGF units. Each SGF unit involves three layers: 1) a feature extraction layer, 2) an event-driven layer, and 3) a training layer. On the top left, there is an example of a four-event classification SGF system: A, B, C, and D are four event types.

In this work, we developed a novel macro-level system titled spike gating flow (SGF) for action recognition as shown in Figure 1. The system consists of multiple SGF units that are connected in a hierarchical manner. An SGF unit consists of three layers: 1) a feature extraction layer for global dynamic feature detection; 2) an event-driven layer for generating event global feature vectors; 3) a supervise-based histogram training layer for online learning (redlines in Figure 1). By employing a dynamic vision sensor (DVS) (Posch et al., 2011)-based gesture dataset (Amir et al., 2017) as a benchmark, the results demonstrated that the developed SGF had great learning performance: 1) the system can achieve the same level of accuracy of 87.5% as the DL but with a training/inference sample ratio of 1.5:1. More importantly, only one epoch is required during the training; 2) to our best knowledge, this is the highest accuracy among the non-BP based SNNs; 3) the system consumes only 9 mW and 99 KB memory resources on an FPGA board at the inference stage. In summary, the contributions are as follows:

- Algorithm aspect: we developed an efficient FSL system for gesture recognition, which behaves like biological intelligence: FSL, energy efficient, and explainable.
- Application aspect: the SGF-based hardware showed reasonable memory size (99 KB) and power consumption (9 mW), which was suitable for the edge/end-device scenarios.
- Learning theory aspect: we concluded one FSL paradigm: 1) a hierarchical structure-based network design involves prior human knowledge; 2) SNNs for global dynamic feature detection.

## 2. The spike gating flow

The Spike Gating Flow (SGF) is a new learning theory to achieve online few-shot training, which is inspired from the neural engineering framework (NEF) (Paulin, 2004) and brain assemble theories (Papadimitriou et al., 2020). In brief, the FSL capabilities rely on the prior knowledge embedded in the hierarchical architecture and global feature computing. The online computing benefits from using dynamic spike patterns to encode both data and control flow. Therefore, different level nodes in the network are served as gates to pass or stop input data information, and spikes are served as gate control signals. We have concluded the key principles of SGF as below:

- **Global feature representations**: Network representations are defined by the combination of different global movement features rather than local pixel features.
- **Tailor designed hierarchical network structure**: A hierarchical structure-based network for conditional data-path execution. Depending on inputs, SGF unit spike patterns are served as gate commands to manipulate data-paths.
- **Histogram based training algorithms**: A global feature-based histogram training adjusts output layer weights based on historical information.

**Figure 2:**
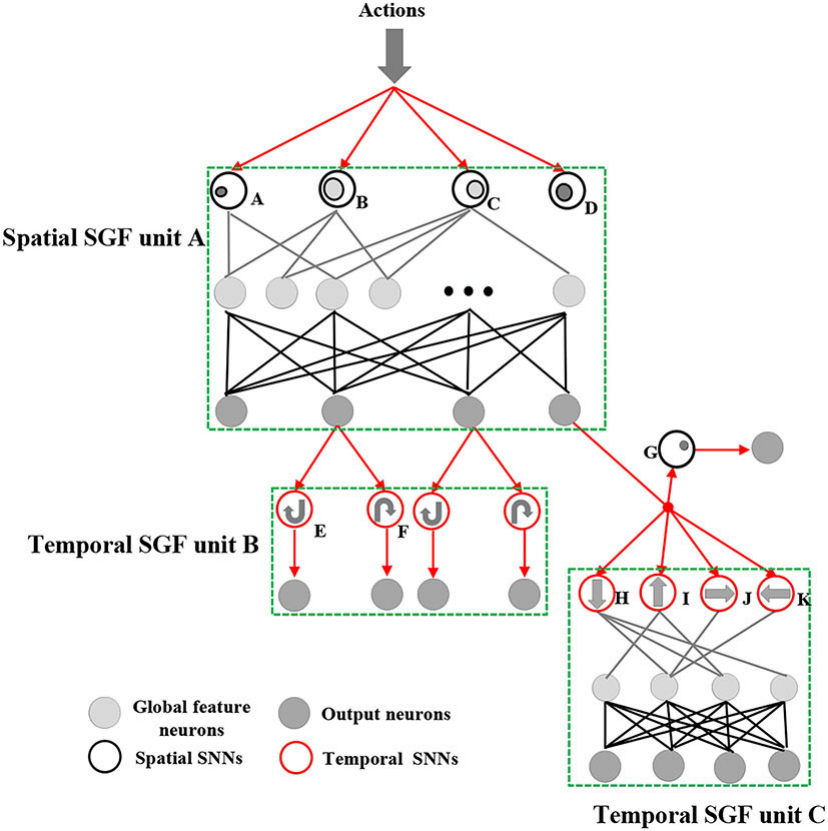
The spiking gating flow (SGF) network architecture. It mainly consists of three SGF units: a spatial SGF unit A and temporal SGF units B and C. A spatial SGF unit A has four SNNs with feature ID index A-D (A: intensive activities at constrained left areas; B: mild activities at plateau left areas; C: mild activities at plateau right areas; D: intensive activities at constrained right areas). A temporal SGF unit B has two SNNs with feature ID E,F (E: clockwise movement; F: counter-clockwise movement). A temporal unit C has four SNNs with feature ID index H-K (H: top-down; I: bottom-up; J: left-right; K: right-left). Also, the developed network has 10 output neurons corresponding to 10 action types.

Based on such principles, we designed three SGF units and carefully connected them into a two-level network, particularly for online gesture recognition. Such an architecture could be considered as a pre-designed learning rule, and each SGF unit was designed based on the cell assemble theories (Müller et al., 2020), and it responded to unique content-based global dynamic features. These units could be assembled into hierarchical levels according to prior human knowledge, which facilitated the learning efficiency of the system. As shown in Figure 2, the top area of the developed network was a spatial SGF unit A, and the bottom areas were temporal SGF units B and C. We designed the SGF network by breaking the complex gesture features into spatial and temporal domains in sequence. For example, the SGF unit A captured spatial features, such as action ranges and intensities, and generated a coarse-grained classification. The SGF units B and C are in charge of refining them into final results by detecting the temporal information. Typically, an SGF unit consisted of three layers: a feature extraction layer, an event-driven layer, and a histogram-based training layer. Also, there can be some structural variations of SGF units. For instance, the SGF unit B has a feature extraction layer only.

For an SGF unit computing process, at first, a feature extraction layer is used to detect global dynamic features of events. Each SGF unit has several corresponding spatial SNNs and temporal SNNs, which target in detecting different global features (features with index A-I are shown in Figure 2). An SGF unit A has four spatial SNN networks, which respond to spatial feature detection as below: A) intensive activities at a constrained left area; B) mild activities at a large left area; C) mild activities at a large right area; D) intensive activities at constrained right areas. An SGF unit B has two temporal SNN networks for E) clockwise movements and F) counter-clockwise movements. Particularly, a prior human knowledge of event sequences is introduced here for designing E and F. An SGF unit C has four temporal SNNs and one spatial SNN for detecting as follows: G) intensive activities at a specific constrained area. H) up-down movements; I) bottom-up movements; J) left-right movements, and K) right-left movements. The detailed computing mechanisms of SNNs are described in Section 3 of SNN design.

Next, there is an event-driven layer that connects SNNs outputs to the global feature neurons. This layer is responsible for generating event feature vectors for the next training layer. Typically, an event class will have several feature vectors types due to the spatiotemporal variations. A feature vector can be defined as a combination of feature indexes of all active SNNs, which are represented by connecting active SNNs to one global neuron. Therefore, for each action type, global feature neuron number is equal to the feature vector type number.

At last, an SGF unit has a fully connected histogram-based training layer, in which each output neuron connects to its all global feature neurons. After each training trail, feature vector histogram will be updated and converted into corresponding weights. In addition, the conversion is a normalization process. This result of the higher the histogram number is, the bigger the weights are. At the inference stage, the feature vector generated from a test sample will be sent into all output neurons for final scores, which follows the equation as below:


(1) $$\begin{array}{l} \begin{array}{ll} S_{m}=\underset{j}{\sum^{\text{​}}}\frac{\sum^{\text{​}}T_{j}^{m}⊙V}{L_{v}}\times w_{j}^{m} & \end{array} \end{array}$$

Where *S*_*m*_∈[0, 1] is a testing sample score at *m*^*th*^ output neuron (*m*^*th*^*classification*); $w_{j}^{m}\in \left[0,1\right]$ is the weight of *j*^*th*^ feature vector for the *m*^*th*^ output neuron; $T_{j}^{m}$ is the *j*^*th*^ feature vector of the *m*^*th*^ output neuron; and *V* is the feature vector of the testing samples. Both the $T_{j}^{m}$ and *V* consist of 1-bit values which belong to {0, 1}. The symbol ⊙ is a bit-wise NXOR operation, and *L*_*v*_ is the length of the feature vector. Then, the final decision is the classification with the highest score. The key advances of such a learning algorithm are that each data sample only requires one training time and tiny computational resources for updating weights, which enables rapid online learning behaviors.

**Figure 3:**
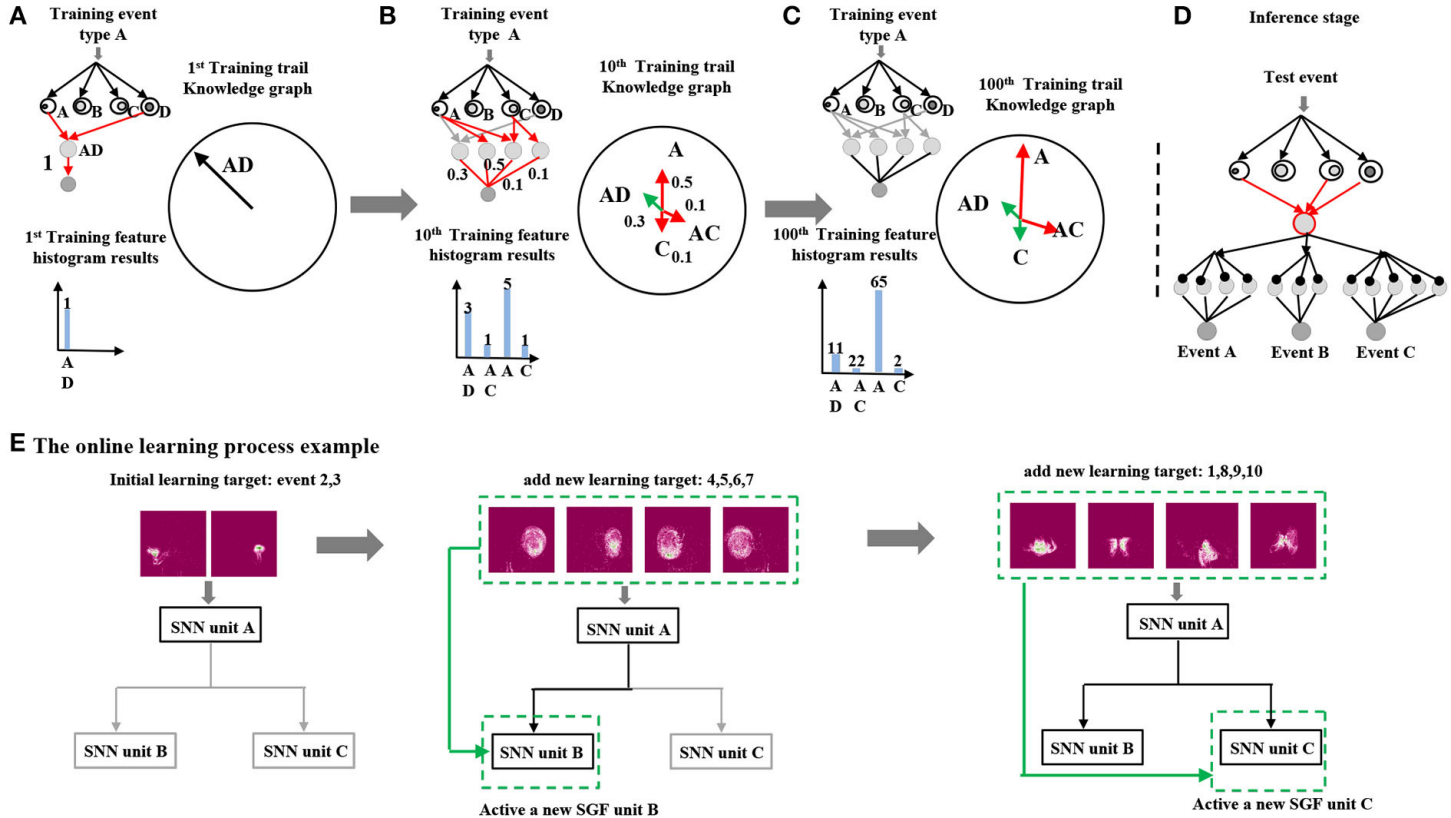
**(A–D)** The histogram-based training example of an event type A. **(E)** An example of the system online learning process.

A detailed example is illustrated in Figure 3. SNNs with feature index *A* and *D* are active at the first training trail, which forms a feature vector[*A*−*D*]. Hence, a corresponding global feature neuron is generated that connects to SNNs with feature indexes *A* and *D* (connected with red lines). A feature vector histogram is also displayed in Figure 3A left. After that, the feature vector[*A*−*D*] histogram values will be converted into output neuron weights of event type *A*. It is clearly seen that the weight is one since there is only one feature vector type (Figure 3A). Meanwhile, a knowledge graph of event type *A* is produced through quantitative analysis of feature vectors distributions (Figure 3A right). At a 10*th* training trail, there are three more feature vectors generated [*A*−*C, A, C*] (Figure 3B left red lines). This indicates that there are in total four types of feature vector in the event type *A*. Identically, corresponding feature vectors histogram numbers [3, 1, 5, 1] will be transformed into event *A* neuron outputs weights *via* a training layer. The feature vector's distribution is also updated in the knowledge graph: a vector with green lines indicates that histogram values are decreased, while a vector with red lines indicates that histogram values are raised. At the end of a 100*th* training trail, there is no new feature vector appeared, which results in the same global feature neuron number as the 10th training trail. The event *A* output neuron weights are updated based on the current histogram numbers as a final result. Similarly, the event types B and C follow the same training procedures.

At the inference stage, a test sample was given into the trained network, which would generate a corresponding test feature vector. It would go through all the output neurons to calculate the final scores. As shown in Figure 3D, there is a trained network which contains three output neurons, whose inference classification result is the maximum one among these output neuron scores.

The online learning process example is shown in Figure 3E. At an initial stage, event group A [3: right-hand wave; 2: left-hand wave] was sent into the network for training. Since event group A contained significant spatial features, only a spatial SGF unit A was active and responsible for generating feature vectors. After finishing learning event group A, event group B [4: right arm clockwise, 5: right arm counter clockwise, 6: left arm clockwise, and 7: left arm counter clockwise] was sent into the network for sequential online learning. Identically, a temporal SGF unit B was active for recognizing clockwise/counter clockwise movements. At last, event group C [1: hand clap, 2: left-hand wave, 8: arm rolls, 9: air drum, and 10: air guitar] was sent into the network that contained complex combinations of vertical and horizontal movements. The SGF unit C was active for learning such features. As it can be seen, the final network architecture varied depending on the learning targets.

## 3. Design of SNNs

We have developed two types of SNNs, spatial SNN and temporal SNN, and a preprocessing module, spatiotemporal (ST) cores. An ST core aims to reduce the background noise of the DVS camera and enhance the key ST information. Spatial SNNs respond to capturing event's spatial features. Temporal SNNs are expert in distinguishing object movement directions. By combining these SNNs, a system can have rich representations of various ST features for action classifications.

### 3.1. ST core

Since a DVS camera has a unique output format, a ST core is designed for DVS output preprocessing: 1) to reduce the DVS output noise and 2) to enhance the key spatiotemporal information. An ST core involves two-stage computations: spatial and temporal processing. The mathematical model is as below:


(2) $$\begin{array}{l} ST_{m}^{t}={\left[\int_{t-\text{Δ}ST_{t}}^{t}{\left[\sum_{i}^{i+\text{Δ}ST_{s}}d_{m}^{t}\right]}^{\theta_{s}}dt\right]}^{\theta_{t}} \end{array}$$

Where ${ST}_{m}^{t}$ is the output of the *m*^*th*^ ST core at frame *t*; $d_{m}^{t}$ is the output of the *m*^*th*^ DVS sensor pixel at frame *t*, which equals to –1 or +1; Δ*ST*_*s*_ is the detection range of an ST core. The function ${\left[S\right]}^{\theta_{s}}$ equals 1 if S is over spatial thresholds θ_*s*_. Regarding the temporal computations, Δ*ST*_*t*_ is an integration window, and θ_*t*_ is a temporal threshold. The function ${\left[T\right]}^{\theta_{t}}$ equals 1 if *T* is over spatial thresholds θ_*t*_. As a result of this, by adjusting the above four parameters [Δ*ST*_*s*_, θ_*s*_, Δ*ST*_*t*_, and θ_*t*_], we can configure the ST core filtering behaviors properly. In general, each SNN will require an ST core for feature extraction. More details of preprocessing the DVS gesture dataset are illustrated in the Result Section 4.3.

### 3.2. Spatial SNNs

A spatial SNN is designed for extracting spatial features based on the ST core outputs. The computing mechanism is quite similar to an ST core. However, the major differences rely on the following: 1) the outputs of a spatial SNN are a feature vector; 2) a spatial SNN does not require temporal information, and it accumulates all the frames of [0, T] together first and performs spatial computing. The spatial SNN equations are as below:


(3) $$\begin{array}{l} \begin{array}{l} \begin{array}{l} SP_{m}={\left[\sum_{i}^{i+\text{Δ}SP_{s}}{\left[\int_{t=0}^{t=T}ST_{m}^{t}dST\right]}^{\theta_{i}}\right]}^{\theta_{a}} \end{array} \end{array} \end{array}$$

**Figure 4:**
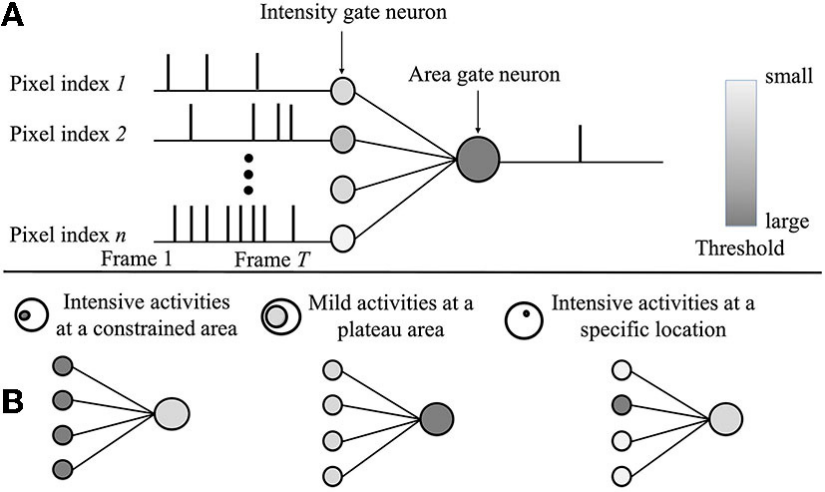
**(A)** The spatial spiking neural network (SNN) computing mechanism. The event consists of T frames in total. **(B)** Examples of three spatial SNNs focus on different global feature detection.

Where *T* is the total frame number of an event. Δ*SP*_*s*_ is the detection size and *SP*_*m*_ is the *m*_*th*_ spatial SNN outputs. The outputs can be a single bit or multiple bits. θ_*i*_ is the gate neuron threshold for intensity, and θ_*a*_ is the gate neuron threshold for the area. As Figure 4A depicts, an event consisting of T frames is processed by intensity gate neurons first. After that, intensity gate neuron spikes are employed as inputs for area gate neurons.

By adjusting two thresholds' values, three different spatial SNNs can be reconstructed in Figure 4B: 1) spatial SNNs with feature index [*A, D*]: intensive activities as a constrained area [θ_1_>θ_2_]; 2) spatial SNNs with feature index [*B, C*]: mild activities at a large area [θ_1_ < θ_2_]; and 3) spatial SNNs with feature index [*G*]: intensive activities at a specific location.

### 3.3. Temporal SNNs

Temporal SNNs are designed for detecting the movement directions of an event (e.g., up-down, left-right). The key principle is to encode the object's temporal location into temporal neuron spiking patterns. The equation is shown below:


(4) $$\begin{array}{l} \begin{array}{l} TE_{m}^{t}={\left[\sum_{i}^{n^{t-\Delta TE_{t}}}{\left[l_{m}^{t}-l_{i}^{t-\Delta TE_{t}}\right]}^{\theta_{l}}\right]}^{\theta_{te}} \end{array} \end{array}$$

Where ${TE}_{m}^{t}$ is the output of the *m*^*th*^ temporal neuron at frame *t*; $l_{i}^{t}$ is the location of the *i*^*th*^ temporal active neuron at frame *t*, the location can be either vertical or horizontal information depending on the temporal SNN type. Δ*TE*_*t*_ is the comparison frame window; θ_*l*_ is the location index threshold, $n^{t-\Delta {TE}_{t}}$ is the active neuron number at frame *t*−Δ*TE*_*t*_. θ_*te*_ is the temporal neuron spiking threshold. For each frame, the object location is calculated by selecting the maximum location index of fired neurons. Therefore, objects' temporal movement patterns can be obtained by a combination of the generated object location at each frame, which serves as event temporal feature vectors.

**Figure 5:**
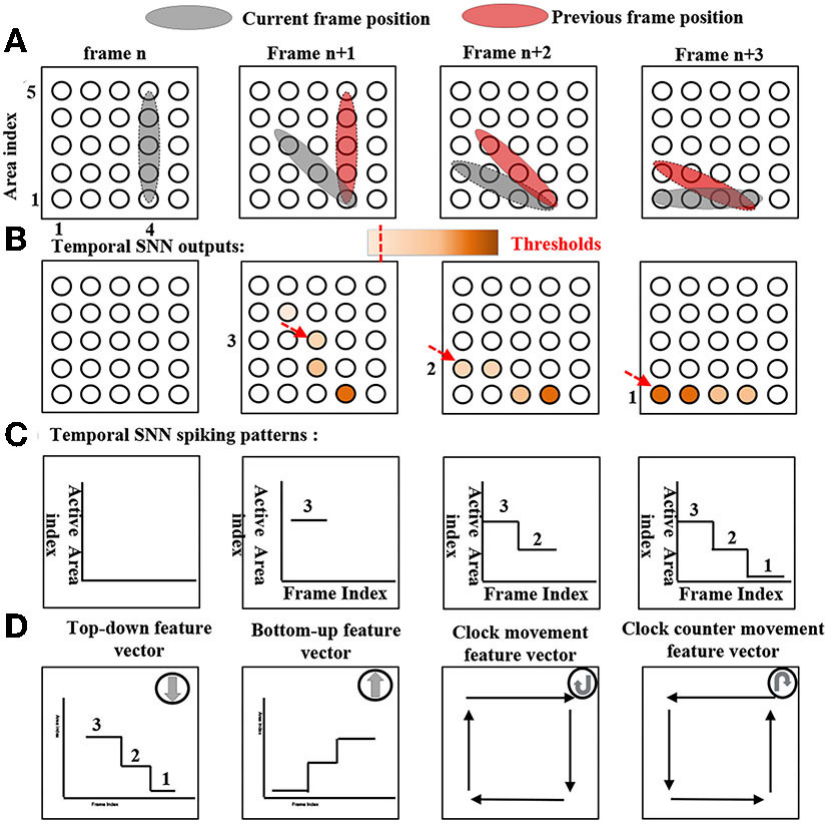
The temporal SNN with feature index H top-down computing mechanism. Also, reference patterns of temporal SNNs with feature indexes E, F, and I are shown at bottom as well. **(A)** The object movement. **(B)** Temporal SNN outputs. **(C)** Detected temporal SNN spiking patterns. **(D)** Reference pattern.

For instance, Figure 5 shows a temporal SNN with feature index *H* for detecting top-down movements. At a frame *n*, an object is vertically located at areas from [4, 1] to [4, 5]. Since this is the first frame, a temporal SNN does not generate activities because there is no reference for comparison. At a frame *n*+1, an object is moving down to a new location as the gray color indicates. Each active neuron (neuron that receives non-zero ST core outputs) compares its location to all the active neurons at the previous compared frame. If the current neuron location is lower than that of the previous frame, the current neuron receives an input value from the compared neuron. In this top-down case, the location index is defined as the vertical information (Y-axis). The temporal SNN outputs are shown in Figure 5B, neurons with dark colors indicate large input values, while neurons with light colors indicate limited input values. Neurons then generate a spike where values are above a threshold θ_*te*_. After calculating all the frames, the object's temporal movements are represented by the generated temporal feature vectors. A temporal SNN with feature index *H* will be active if an object movement temporal feature vector is consistent with its reference feature vector which is shown in Figure 5D bottom (top-down feature vector). Identically, temporal SNNs with feature index [*I, J, K*] follow the same computing mechanisms. Corresponding reference feature vectors are also shown in Figure 5D bottom. Reference feature vectors that can be pre-defined or learned depend on the task. Particularly, SNNs with feature index [*E, F*] clockwise and counter clockwise require temporal feature vector timing information that is based on a prior human knowledge. The clockwise counter event temporal pattern sequence is defined as [top-down, left-right, bottom-up, and right-left], and the clockwise event temporal pattern sequence is defined as [top-down, right-left, bottom-up, and left-right].

## 4. Results

**DVS Dataset:** A dynamic vision sensor gesture dataset (Amir et al., 2017) (10 classes of gesture actions of which each contains 98 training sequences and 24 test sequences) is employed to verify the system's performance. The event-driven sensor data is different from the traditional video since the DVS records the change in each pixel on a 128^*^128 canvas independently. Specifically, a spike will be recorded into sequences if the brightness is changed in the pixel (with the column and row address).

**Experiment Setting:** We preprocessed the event-driven sensor data with a standard method (Rebecq et al., 2019). A frame is defined to be 1,000 continuous spikes, and each sequence in the dataset is divided into 50–80 frames. For each class, we used 36 sequences for training and 24 sequences for testing.

### 4.1. Network architecture

The network details and statistical results are shown in Table 1. The number of input and output neurons of our designed SNNs can be flexibly configured. The SNNs in unit B have the largest number of input (16,384) and output (160) neurons because of the more complex task. As a result, unit B has more operations (2.1 M) than unit A/C (7–63 K). However, they have the similar model size since the unit B does not have the event layer and the histogram-based layer. The details of parameter calculation are described in Appendix A.

**Table 1:** The developed network architecture information.

**SGF**	**Unit A**				**Unit B**		**Unit C**				
SNN index(Type^*^)	**A(*SP*)**	**B(*SP*)**	**C(*SP*)**	**D(*SP*)**	**E(*TE*)**	**F(*TE*)**	**G(*SP*)**	**H(*TE*)**	**I(*TE*)**	**J(*TE*)**	**K(*TE*)**
Input neuron number	Unit A shares 1,764 input neurons				Unit B and C share 16,384 input neurons						
Output neuron number	16	18	18	16	160	160	2	2	2	2	2
Feature vector length	16	18	18	16	160	160	2	2	2	2	2
Number of OPs	56.0 K	63.0 K	63.0 K	56.0 K	2.1 M	2.1 M	7.0 K	27.2 K	27.2 K	27.2 K	27.2 K
Model size	304 Byte	342 Byte	342 Byte	304 Byte	240 Byte	240 Byte	2 Byte	16 Byte	16 Byte	16 Byte	16 Byte

*SP indicates the spatial SNN and *TE* indicates temporal SNN.

### 4.2. System accuracy

In Table 2, we first compared the developed network with two typical SNN-based gesture recognition networks, an STDP-based SNN (George et al., 2020) and an SGD-based SNN (Perez-Nieves et al., 2021). To the best of our knowledge, our system achieved the highest accuracy of 87.5% among state-of-the-art non-BP-based SNNs. Regarding ANN/DNN converted SNN, the developed network can reach the same level of accuracy as SLAYER (Shrestha and Orchard, 2018), but slightly lower than ConvNet (Amir et al., 2017) at 96.5%, SCRNN (Xing et al., 2020) at 96.59%, Converted SNN (Kugele et al., 2020) at 96.97%, and PointNet++ (Qi et al., 2017) at 97.08%. However, the network model size can be reduced by 456 times compared to the ConvNet (Amir et al., 2017), and the number of operations can be reduced by 53 times compared to the PointNet++ (Qi et al., 2017).

**Table 2:** The comparison between state-of-the-art methods and the proposed spiking gating flow (SGF) network.

**Name**	**Type**	**Learning**	**Learning**	**Model information**				**Training cost**		**Accuracy**
		**method**	**style**	**Size**	**Diff(×)**	**OPs**	**Diff(×)**	**Epoch**	**T/I ratio**
Reservoir CSNN (George et al., 2020)	SNN	STDP	Offline	3.17 MB	88.7↑	-		-	3.8:1	65.0%
Heterogeneity Network (Perez-Nieves et al., 2021)	SNN	SGD	Offline	125 KB	3.4↑	-		-	3.8:1	82.1%
SLAYER (Shrestha and Orchard, 2018)	SNN	BP	Offline	1034.8KB	28.3↑	79.8M	9.6↑	739	3.8:1	93.64%
SCRNN (Xing et al., 2020)	ANN2SNN	BPTT	Offline	732.34KB	20.0↑	81.91 M	9.9↑	100	4.1:1	96.59%
Converted SNN (Kugele et al., 2020)	ANN2SNN	BP	Offline	500KB	13.7↑	651 M	78.7↑	10	3.8:1	96.97%
ConvNet (Amir et al., 2017)	DNN2SNN	BP	Offline	16.3 MB	456↑	946.82 M	114↑	250	3.8:1	96.5%
PointNet++ (Qi et al., 2017)	DNN2SNN	BP	Offline	3.50MB	98↑	440.0 M	53.2↑	250	3.8:1	97.08%
**This work**	**SNN**	**SGF**	**Online**	**36.58 KB**		**8.27 M**	**1**	**1.5:1**	**87.5%**

“-” Indicates the data can not be calculated or not mentioned in the corresponding paper. Bold indicate the results in our method. ↑ Indicates that our method has improvement compared to the related works.

Finally, the developed SGF only requires 1 training epoch at a condition of training/inference ratio of 1.5:1, while DL networks typically require hundreds of training epochs at a condition of 3.8:1. This indicates that the system training cost is significantly lower than the DL-based networks.

### 4.3. SNNs behaviors

The spiking neural networks can be tuned by adjusting the thresholds. For a particular dataset, we employed a grid search method to find an optimal threshold based on the training dataset and required functionalities.

**Figure 6:**
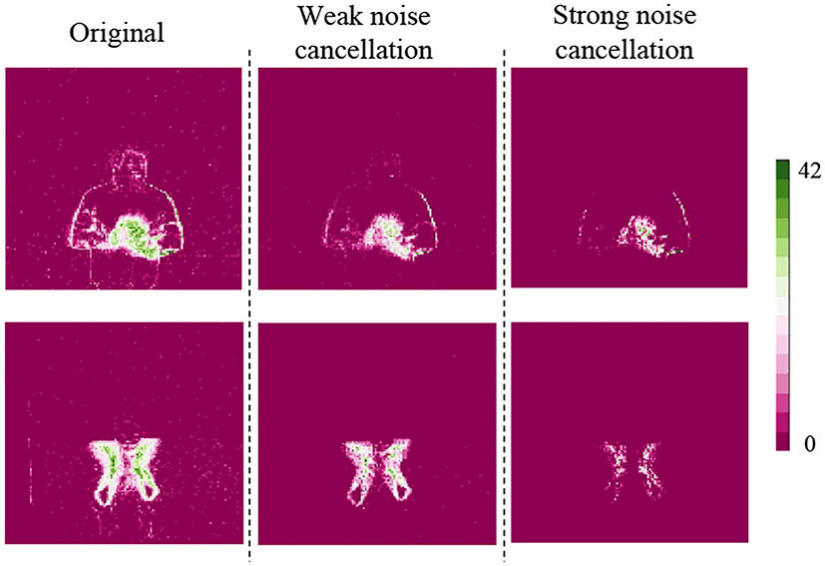
The spatiotemporal (ST) core weak and strong noise cancellation results.

The spatiotemporal core noise cancellation performance is shown in Figure 6. At the left side of the figure, there are original event pictures of a hand clap and an air drum. Event pictures are obtained *via* an ST core model process. A color bar on the right displayed spike intensities at each pixel. In the middle, there are the results of an ST core with weak noise cancellation (parameters are 1, 1, 2, 2), it is clearly seen that most of the sparse noise are disappeared. A right, there are results of an ST core with strong noise cancellation (parameters are 1, 1, 6, and 5), only most significant features are kept at this case.

**Figure 7:**
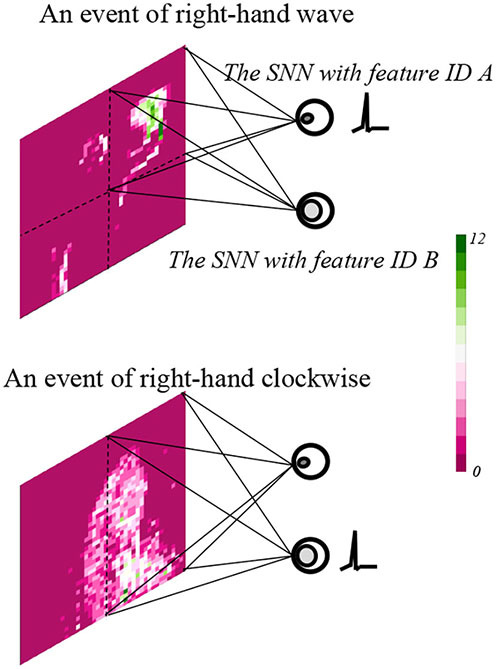
The spatial SNN with feature indexes A and B results on events of the right-hand wave and right-hand clockwise.

Spatial SNNs computing performance is shown in Figure 7, events of the right-hand wave and the right-hand clock-wise movement are employed as examples. The results indicate that SNN with feature index A generates a spike for an event of the right-hand wave, since it is only sensitive to intensive activities in a constrained area. Furthermore, the SNN with feature index B generates a spike for an event of the right-hand clockwise, which results from the interest of mild activities on a large area.

**Figure 8:**
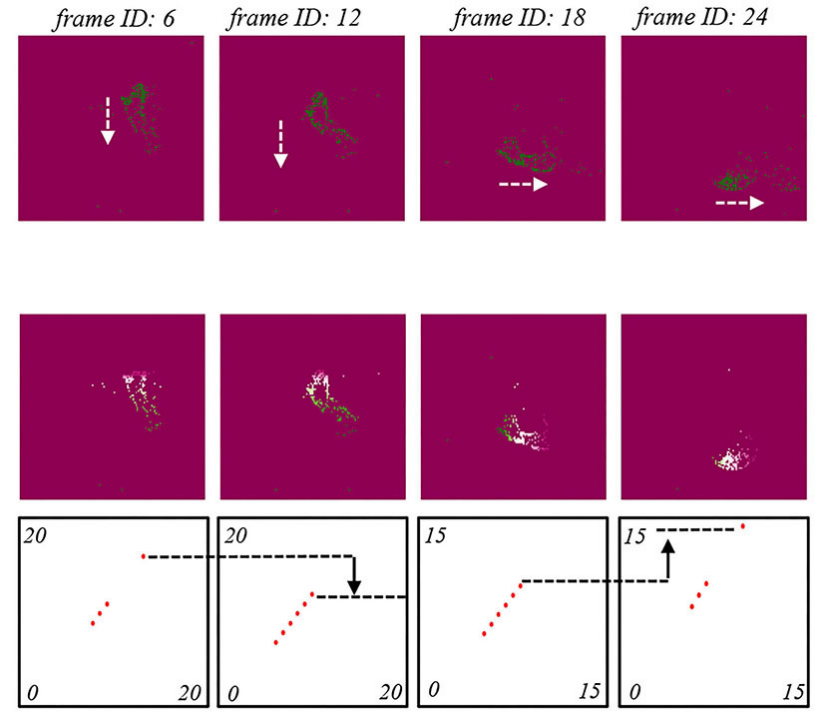
The temporal SNN with the performances of feature index E performances. The origin frames of a counter-clockwise counter event. The temporal SNN outputs and spiking patterns. The temporal SNN with feature index H. The temporal SNN with feature index J. The arrow indicates moving trajectory of the generated spikes.

Temporal SNNs computing performance is shown in Figure 8. Here, we employed an event of clockwise counter as an example. Four original frame information is shown in the top of Figure 8 top. Corresponding temporal SNNs with feature indexes H (top-down) and J (left-right) outputs are shown in the middle of Figure 8. It was clearly observed that there was a top-down movement pattern followed by a left-right movement pattern, which were identical to the pattern sequence of clockwise counter event.

### 4.4. FSL performance

**Figure 9:**
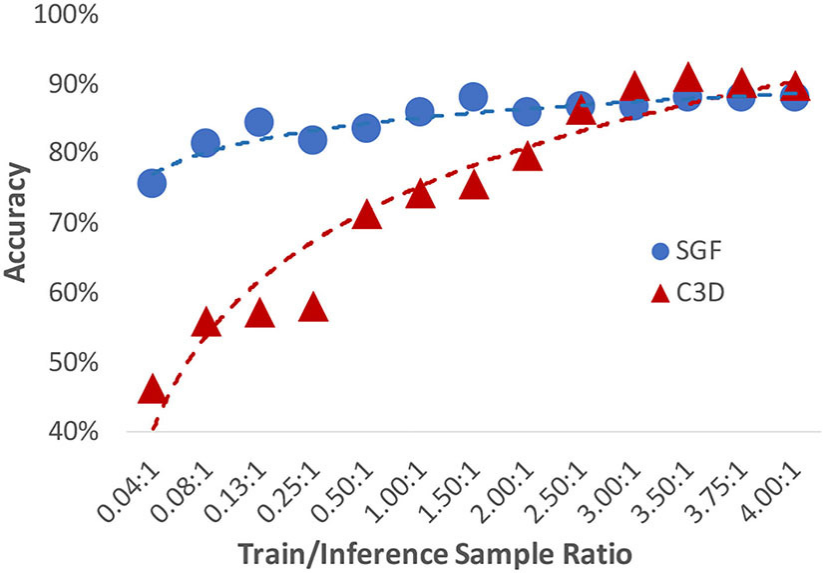
A comparison of the few-shot learning (FSL) performances on both SGF and C3D networks.

We also investigated the system's FSL performance. By varying the data ratio between the training and inference stage, results are shown in Figure 9. Compared to a typical action recognition deep learning model C3D (Tran et al., 2015) (red line), the developed SGF illustrated excellent FSL performances. At a training/inference data number ratio 1.5:1 condition, the SGF reached the highest accuracy of 87.5%, while the C3D network only has 70% accuracy. However, there is a cross-point at a training/inference data number ratio of 3.8:1. The C3D network reached above 90% accuracy and surpassed the SGF network. In summary, we concluded the key design principles of the developed FSL paradigm: 1) a hierarchical structure-based network design involves prior human knowledge; 2) SNNs for global dynamic feature detection.

### 4.5. Design quality of SNNs

**Figure 10:**
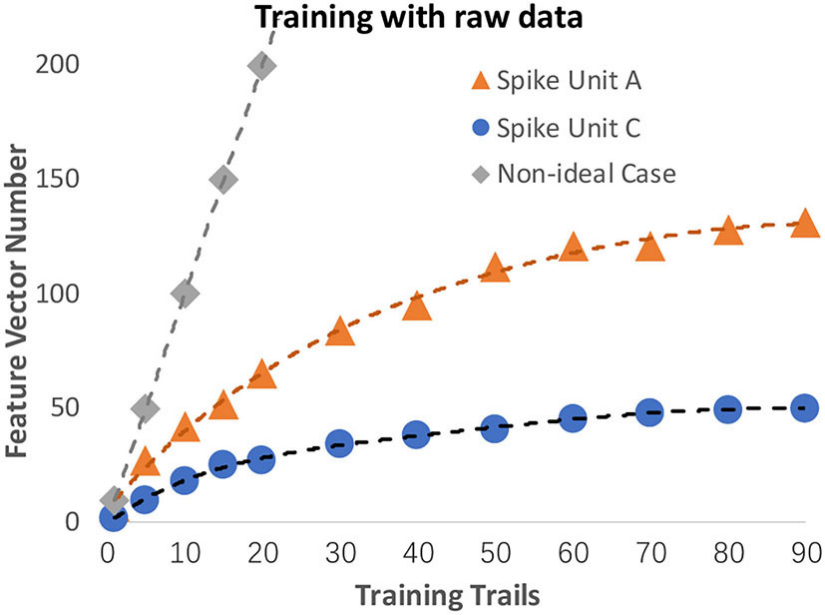
The SNNs design qualities performances. The SGF units A and C are shown in orange and blue color, while a non-ideal design case is shown in gray color.

We evaluated our SGF unit design by visualizing the convergence speed. The faster the convergence speed, the higher the design qualities. In each trail, a training sequence was used to update the feature vectors. For an effective design, the number of feature vectors should be converged as the number of training trails increases. Thus, we visualized the number of generated feature vectors in Figure 10. From the experiment, we can draw several conclusions: a) Our SGF units A and C are feasible because the number of feature vectors is gradually convergent as we expected. For comparison, in the non-ideal case, the number of feature vectors increased linearly with the number of training trails so that cannot converge. b) Our SGF units A and C have good FSL potential. The results indicate that after 40 trails, the SGF units A and C already converged.

### 4.6. Hardware implementations

**Figure 11:**
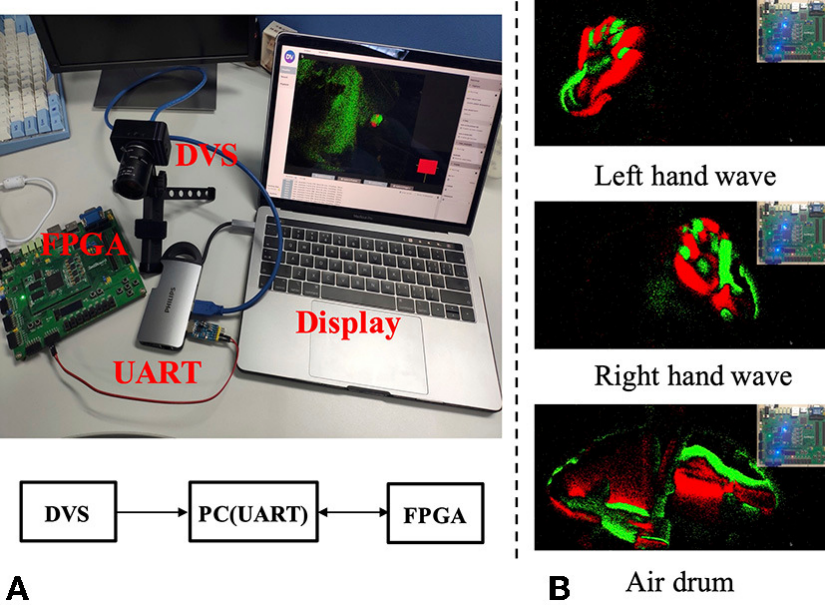
**(A,B)** The experimental setup: an SGF inference model is implemented on an FPGA ZedBoard XC7Z020-CLG484-1 for testing system hardware performances.

A spiking gating flow inference model is implemented on an FPGA ZedBoard XC7Z020-CLG484-1 for testing the performance of a system hardware performance. As a proof of the concept, the hardware implemented SGF has the capability to classify five events. The developed hardware architecture is largely based on Luo et al. (2016) with modifications. The system configuration is shown in Figure 11A, a DVS camera DAVIS346 (Inivation) is directly connected to a laptop HP Pro Book 430 G6 *via* a custom designed UART communication protocol. Three event types classification results are shown in Figure 11B: left wave, right wave, and air drum. The power consumption is 9 mW and memory size is 99 KB, which means our SGF can serve as an ideal candidate for edge/end-device applications.The detailed hardware implementations are shown in Appendix B.

## 5. Discussion

This work presents a novel system titled SGF which has a strong dispersity with the current mainstream DL networks. First, we employed a standard DVS gesture classification as a proof of concept. Regarding the training performances, the developed network can achieve the same level of accuracy with the DL under a condition of the training/inference data ratio 1.5:1. More importantly, only one training epoch is required during the learning periods, which significantly reduced the system training cost. Also, in terms of the model complexity, the SGF model size is approximately 379 times smaller than a standard CNN network, and 2.8 times smaller than an SNN. Then we implemented a developed SGF inference model on a tailor designed hardware resulting limited power consummations (9 mW) and memory resources (99KB). At last we draw conclusions of essential factors for achieving few-shot learning paradigms: 1) a hierarchical architecture design encoded with human prior knowledge; 2) SNNs for global feature detections. At last, although the system capability has a considerable distance compared to the current DL network, the system shows the essential biological intelligence (e.g., few-shot learning, energy efficient, explainable) at a particular scenario. This may inspire us to design the next generation DL algorithms, and also raise a wider discussion among groups of computer hardware architecture, neuroscience and algorithms.

One of the major future works is that the network does not have strong generalization capabilities, which may not be suitable for processing a large-scale dataset (Soomro et al., 2012) (e.g., UCF101). This is due to the network architecture being tailor-designed for the gesture recognition task. Such a design principle of fusing prior human knowledge with SNNs global feature detection introduces a biological intelligence-based system but with limited flexibility. In the future, we will focus on solving the generalization issue in various technology paths: 1) designing an SNN based network architecture search (NAS) mechanism which is similar to the Auto ML (He et al., 2021); 2) introducing a reinforcement-learning based agent to generate learning rules that equal to the prior human knowledge (Williams, 1992); 3) utilizing biological brain assembling theories to build a learning logic-based architecture (Papadimitriou et al., 2020; Wu et al., 2022).

## Data availability statement

Publicly available datasets were analyzed in this study. This data can be found at: https://research.ibm.com/interactive/dvsgesture.

## Author contributions

ZZ and JL finish the main part of this work software, hardware, methodology, and writing. All other authors finish the remain work experiment, reviewing, and editing.

## Funding

This work was supported by the project of National Key Research and Development Plan under Grants No. 2018YFB2202604 and the Science and Technology Commission of Shanghai Municipality under Grants 2018SHZDZX01.

## Conflict of interest

Authors TX, FT, and JZ are employed by Alibaba Group. Authors YX and JL are employed by Alibaba DAMO Academy. The remaining authors declare that the research was conducted in the absence of any commercial or financial relationships that could be construed as a potential conflict of interest.

## Publisher's note

All claims expressed in this article are solely those of the authors and do not necessarily represent those of their affiliated organizations, or those of the publisher, the editors and the reviewers. Any product that may be evaluated in this article, or claim that may be made by its manufacturer, is not guaranteed or endorsed by the publisher.
