# Supplementary material for: The spike gating flow: A hierarchical structure-based spiking neural network for online gesture recognition
Source: Front Neurosci. 2022 Nov 2;16:923587. doi: 10.3389/fnins.2022.923587 (PMC9667043; doi:10.3389/fnins.2022.923587)
Supplement: Supplementary file 1 [file Data_Sheet_1.ZIP › Appendix/SGF_supplementary_material.pdf]

frontiers

Supplementary Material

1 NETWORK DETAILS

1.1 SGF

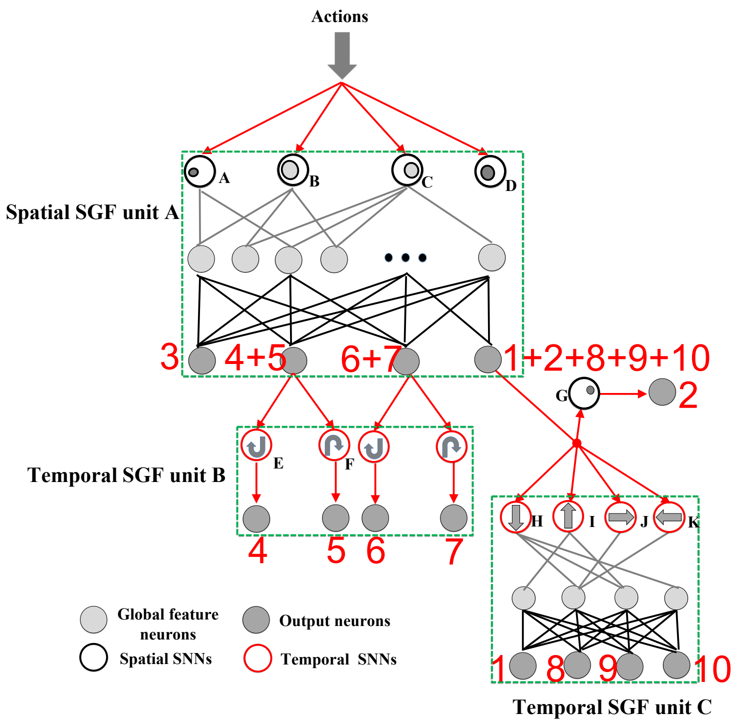

Figure S1. The detailed architecture of SGF network.

The detailed structure of SGF is shown in Fig. S1, the red number of output neurons indicate different event types:

- 1: hand clap;
- 2: left hand wave;
- 3: right hand wave;
- 4: right arm clockwise;
- 5: right arm counter clockwise;
- 6: left arm clockwise;
- 7: left arm counter clockwise;
- 9: air drum
- 10: air guitar.

At first, SGF unit A classifies actions into four types: 3, 4+5, 6+7 and 1+2+8+9+10. Then SGF unit B detects action types of 4, 5, 6 and 7. Finally, SGF unit C responses for the action 1, 2, 8, 9 and 10.

The network parameter calculation is shown in Tab. S1. Spatial SNNs with feature index A/D, B/C and G have the sub-network number 16, 18 and 2, respectively. And temporal SNNs with feature index E/F and

Layer	Calculation	Number	Model size
ST core 1	42*42*2*8/8	1	3528 Byte
ST core 2	128*128*2*8/8	1	32768 Byte
A/D	(110+2*8)/8=19	16	304 Byte
B/C	(110+2*8)/8=19	18	342 Byte
G	2*8/8	2	4 Byte
E/F	3*8/8	160	480 Byte
H/I/J/K	8/8	2	32 Byte
		Total	36.58KB

Table S1. The details of SGF parameter calculation.

Layer	Calculation	Number	Operation number
ST core 1	3*3*42*42*80+42*42*80	1	1.35 MOPs
ST core 2	1*1*128*128*80+128*128*80	1	2.5 MOPs
A/D	42*42+(42*42-1)+1	16	56.0 KOPs
B/C	42*42+(42*42-1)+1	18	63.0 KOPs
G	42*42+(42*42-1)+1	2	7 KOPs
E/F	(30*30+30)*3*10	160	4.26 MOPs
H/I/J/K	(30*30+30)*3*10	2	54.5 KOPs
		Total	8.27 MOPs

Table S2. The details of SGF operation number calculation.

H/I/J/K have the sub-network number 160 and 2. Also, the memory for ST core to store the neuron state is counted as the model size cost, as shown in Tab. S1 first two row. As we can see, the neuron state for ST core takes the largest part of memory consumption, while the feature vector storage is relatively small. Also, Operation number of different networks are displayed in the Tab. S2.

IC	OC	K	S	Pad	H/W(out)	Parameters	MACs
6	12	3	2	0	31	648	622728
12	252	4	2	0	14	48384	9483264
252	256	1	1	0	14	64512	12644352
256	256	2	2	0	7	262144	12845056
256	512	3	1	1	7	1179648	57802752
512	512	1	1	0	7	262144	12845056
512	512	1	1	0	7	262144	12845056
512	512	1	1	0	7	262144	12845056
512	512	2	2	0	3	1048576	9437184
512	1024	3	1	1	3	4718592	42467328
1024	1024	1	1	0	3	1048576	9437184
1024	1024	1	1	0	3	1048576	9437184
1024	1024	2	2	0	1	4194304	4194304
1024	1024	1	1	0	1	1048576	1048576
1024	968	1	1	0	1	991232	991232
968	2640	1	1	0	1	2555520	2555520
					Total	18995720	211501832
					Total	18.12M	201.70M

Table S3. The process of parameters and MACs of ConvNet.

1.2 ConvNet

The layer-by-layer computation of parameter and operation number is illustrated in the Table S3 (1).

Layer type	N	c/IC	#Parameters	#MACs	
GAT	1024	1024	1024	349525.3333	715827882.7
GAT	384	1024	1024	349525.3333	184549376
GAT	128	1024	1024	349525.3333	50331648
MLP	64	1024	512	524288	33554432
MLP	64	512	256	131072	8388608
MLP	64	256	10	2560	163840
			Total	1706496	992815786.7
			Total	1.63M	946.82M

Table S4. The process of parameters and MACs of PAT network.

1.3 PAT

The PAT (2) are formed with 3 sequential GAT and 3 MLP as shown in Table S4.

2 HARDWARE IMPLEMENTATION

The appendix 2 presents a conceptual hardware architecture for implementing an SGF inference model (five events classifications).

2.1 AER Bus

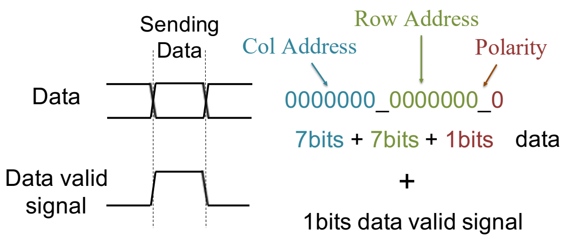

Figure S2. The timing diagram of AER signals.

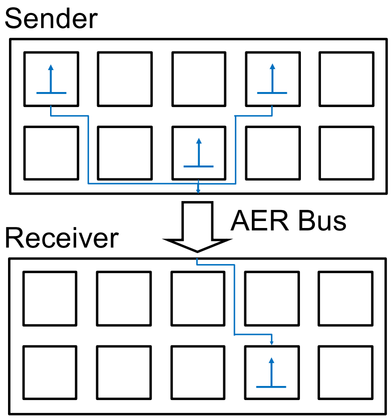

Figure S3. Schematic diagram of AER communication between modules.

In the hardware implementation, the spike based information is transferred among different modules via an Address-Event Representation (AER) communication protocol (3).

As depicted in Fig. S2, the AER data consists of column/row addresses of the source neuron and the polarity of a spike event. In practice, we choose 7-bit for both column and row address, and 1-bit for the polarity. Therefore there are total 15 bits to represent the location and polarity information of a spike. Fig. S3 shows how the sender module and the receiver module is communicated by the AER bus. When a spike event is released from an sender module, the output spikes will be packed and push in the AER sending FIFO. In the meantime, the AER bus continuously transmits the data package when the AER sending FIFO is not empty. The destination address is calculated by the receiver module via a look-up table module.

2.2 Hardware architecture

An overview of the system is shown in Fig. S4. The system configuration is as below: a PC controller sends configuration commands (e.g. network architecture parameters, threshold parameters) to the FPGA based SGF system, and receives the inference result from the system via a Universal Asynchronous Receiver/Transmitter (UART) protocol. Also, A DVS camera is linked to the SGF system to provide

real-time event data. The data paths are labeled as blue lines while the control signals are indicates by the red lines.

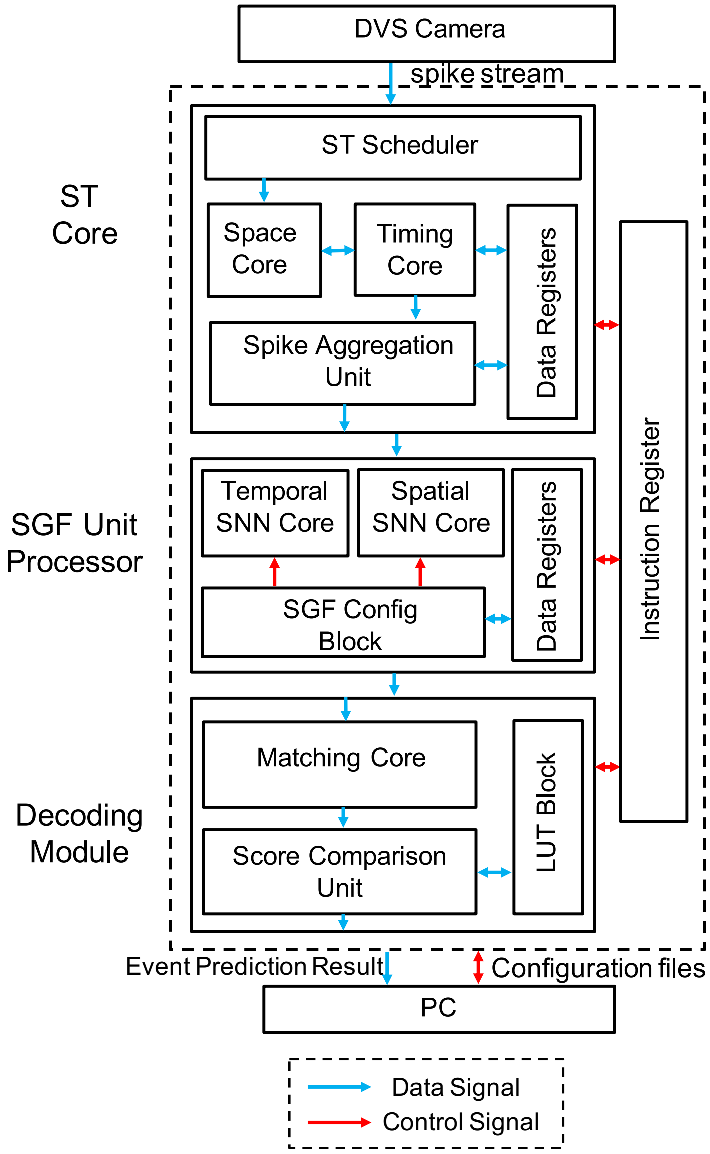

Figure S4. An overview of the SGF hardware system.

The Spatial-Temporal (ST) core module consists of a Space core (S core) and a Timing core (T core), which response for integrating spikes in the space and time domain. And data registers are employed to save the neurons' status. Also a ST scheduler is implemented to translate the input AER data addresses to the corresponding ST core destination addresses.

In the SGF unit processor, there are four main components: a temporal SNN cores, a spatial SNN core, a SGF configuration block and data registers. SNN cores can implement several SNNs in a time multiplexing manner. At the initialization stage, a SGF configuration block receives the network architecture information from an instruction register. A SGF network is constructed by combining multiply temporal and spatial SNNs that specifically followed the instructions. The output spikes of SNNs are formed into feature vectors for the next stage processing.

The decoding module is to generate the final inference result. Feature vectors that generated at the training stage are stored in the Look-Up-Table (LUT) block. When the decoding module receives a test feature vector from a SGF unit processor, the matching core will calculate final scores based on the implemented algorithm. After a calculation of all the event types' score, the maximum one will be chosen as the predict result via a score comparison unit.

REFERENCES

1 .Amir A, Taba B, Berg DJ, Melano T, McKinstry JL, di Nolfo C, et al. A low power, fully event-based gesture recognition system. *2017 IEEE Conference on Computer Vision and Pattern Recognition, CVPR 2017, Honolulu, HI, USA, July 21-26, 2017* (IEEE Computer Society) (2017), 7388–7397. doi:10.1109/CVPR.2017.781.

2 .Yang J, Zhang Q, Ni B, Li L, Liu J, Zhou M, et al. Modeling point clouds with self-attention and gumbel subset sampling. *IEEE Conference on Computer Vision and Pattern Recognition, CVPR 2019, Long Beach, CA, USA, June 16-20, 2019* (Computer Vision Foundation / IEEE) (2019), 3323–3332. doi:10.1109/CVPR.2019.00344.

3 .Boahen K. Point-to-point connectivity between neuromorphic chips using address events. *IEEE Transactions on Circuits and Systems II: Analog and Digital Signal Processing* **47** (2000) 416–434. doi:10.1109/82.842110.
